# Characteristics of Critically Ill Patients with COVID-19 Compared to Patients with Influenza—A Single Center Experience

**DOI:** 10.3390/jcm10102056

**Published:** 2021-05-11

**Authors:** Frank Herbstreit, Marvin Overbeck, Marc Moritz Berger, Annabell Skarabis, Thorsten Brenner, Karsten Schmidt

**Affiliations:** Department of Anesthesiology and Intensive Care Medicine, Faculty of Medicine and University Hospital Essen, University Duisburg-Essen, Hufelandstr. 55, 45147 Essen, Germany; marvin.overbeck@uk-essen.de (M.O.); marc.berger@uk-essen.de (M.M.B.); annabell.skarabis@uk-essen.de (A.S.); Thorsten.Brenner@uk-essen.de (T.B.); karsten.schmidt@uk-essen.de (K.S.)

**Keywords:** COVID-19, SARS-CoV-2, Influenza, intensive Care, ECMO

## Abstract

Infections with SARS-CoV-2 spread worldwide early in 2020. In previous winters, we had been treating patients with seasonal influenza. While creating a larger impact on the health care systems, comparisons regarding the intensive care unit (ICU) courses of both diseases are lacking. We compared patients with influenza and SARS-CoV-2 infections treated at a tertiary care facility offering treatment for acute respiratory distress syndrome (ARDS) and being a high-volume facility for extracorporeal membrane oxygenation (ECMO). Patients with COVID-19 during the first wave of the pandemic (*n* = 64) were compared to 64 patients with severe influenza from 2016 to 2020 at our ICU. All patients were treated using a standardized protocol. ECMO was used in cases of severe ARDS. Both groups had similar comorbidities. Time in ICU and mortality were not significantly different, yet mortality with ECMO was high amongst COVID-19 patients with approximately two-thirds not surviving. This is in contrast to a mortality of less than 40% in influenza patients with ECMO. Mortality was higher than estimated by SAPSII score on admission in both groups. Patients with COVID-19 were more likely to be male and non-smokers than those with influenza. The outcomes for patients with severe disease were similar. The study helps to understand similarities and differences between patients treated for severe influenza infections and COVID-19.

## 1. Introduction

Infections with the severe acute respiratory syndrome coronavirus 2 (SARS-CoV-2) [1] were first discovered in China [2,3], but rapidly spread worldwide during the first wave of the COVID-19 pandemic in the spring of 2020. While most patients experience only mild symptoms, some develop serious disease. Approximately one third of the patients treated for COVID-19 have to be admitted to the intensive care unit (ICU) [4]. These patients are a serious burden on healthcare systems globally [5,6], exhausting ICU resources in many countries and straining providers [7]. A substantial proportion requires mechanical ventilation and support with extracorporeal membrane oxygenation (ECMO) with data on ECMO use in COVID-19 patients being variable and probably reflecting availability and local protocols [8,9,10,11].

In the past, seasonal waves of viral disease leading to large numbers of cases needing ventilatory support up to ECMO have been observed in patients infected with the influenza virus. Belonging to the family of Orthomyxoviridae, influenza viruses comprise four species (Influenza A, B, C and D virus) with Influenza A and B circulating among humans. 5–15% of the population contract influenza in a typical year with up to 650,000 annual deaths globally. Circulating strains vary annually, altering the effectiveness of vaccinations with vaccines needing adjustments for every flu season [12].

The COVID-19 pandemic has been a matter of public debate and infections with SARS-CoV-2 were compared to the flu by influential politicians [13,14].

Similarities between influenza and COVID-19 are evident with both being infectious viral diseases sometimes requiring ICU admission, ventilatory support up to ECMO assistance [15,16,17,18]. However, unique pathophysiological and clinical features of COVID-19 were described early in the pandemic evolving into specific treatment recommendations.

We compared the characteristics of COVID-19 patients treated in ICU at our tertiary care referral center during the first wave of the pandemic to an equal number of patients with influenza since 2016.

## 2. Materials and Methods

We conducted a single-center retrospective study analyzing COVID-19 patients treated at the ICU of the department of anesthesiology and intensive care medicine, University Hospital Essen, Essen, Germany, during the first wave of the pandemic between January and July 2020. We compared these to patients with influenza and treatment in the same ICU between 2016 and 2020. Only patients with confirmed infection by either virus were included. Detection was performed with polymerase chain reaction (PCR) for SARS-CoV-2 or influenza A/B. Samples were taken as nasopharyngeal swab in non-intubated patients. A bronchioalveolar lavage with virus PCR was performed in all intubated patients.

The study was approved by the ethics committee of the University Hospital Essen (approval number 20-9368-BO). The requirement for informed consent was waived due to the exploratory and retrospective nature of the analysis.

Patient groups were compared regarding baseline characteristics. The main clinical endpoints were mortality and length of stay in ICU. Secondary endpoints were any bacteremia, the incidence of invasive aspergillosis, the need for invasive ventilation, ECMO, and dialysis.

The Charlson Comorbidity Index (CCI) was used to categorize preexisting conditions.

The New Simplified Acute Physiology Score (SAPSII) [19] was assessed on admission to quantify severity of disease.

### 2.1. Hospital Setting

The University Hospital Essen is a tertiary care medical center. The ICU operated by the department of anesthesiology and intensive care medicine is part of the West German Center for Infectious Diseases. All patients are treated in single bed rooms. All rooms are equipped with advanced isolation features, enabling a negative pressure environment in cases of airborne transmissible disease. The department is a referral center for ARDS and ECMO with more than 100 ECMO procedures per year. A substantial proportion of patients is transferred from referring hospitals having been treated in ICU at the referring facility. The department offers a critical care transport system with a mobile ECMO unit enabling initiation of ECMO at the referring hospital and transfer of the patient with or without ECMO via ground or air ambulance. The ICU has a 24-h coverage by final year residents in three shifts and an attending with board certification “intensive care medicine” on site during the day and on call at night. During the day, two fellows in “intensive care medicine” are present. ECMO referrals are performed by a team of two physicians with one having a board certificate “intensive care medicine” and two EMT/paramedics.

### 2.2. Patient Population

#### 2.2.1. COVID-19 Cohort

All patients admitted to ICU with a confirmed infection with SARS-COV-2 were included during the first phase of the pandemic (January 2020–July 2020). A total of 64 patients infected with SARS-CoV-2 were included. Of these, 43 patients were transferred from other facilities and 21 patients were admitted via the emergency department or the infectious diseases department.

#### 2.2.2. Influenza Cohort

All patients admitted to ICU with a confirmed infection with influenza A/B from October 2016 to March 2020 flu seasons were included. A total of 64 patients with influenza were included. Of these, 39 patients were transferred from referring hospitals and 25 were admitted via the infectious diseases or the emergency department.

The patient population is depicted in Table 1:

### 2.3. Treatment Protocols

#### 2.3.1. General Treatment

All patients admitted to the ICU were subject to a standardized treatment protocol consisting of extensive laboratory testing on admission, microbiological testing, invasive monitoring, and ultrasound exam. The initial protocol is outlined in Table 2.

The decision to perform endotracheal intubation and invasive ventilation was at the discretion of the treating intensivist.

Invasive ventilation was standardized to a tidal volume of 6 mL/kg ideal body weight with a PEEP above the lower inflection point obtained using a low flow pressure/volume curve (Dräger Evita V500, Dräger, Lübeck, Germany) and a driving pressure below 15 mbar.

A prone position trial was performed in all cases of moderate or severe ARDS with a duration of 16 h [20]. Prone position was continued for at least three days in responders (defined as improved PaO_2_/FiO_2_, improved dynamic compliance).

Inhaled nitric oxide (NO) was applied (NO-A applicator, EKU, Leiningen, Germany) in all cases of moderate or severe ARDS with pulmonary artery hypertension assessed with echocardiography or pulmonary artery catheter. NO was discontinued in patients not showing improvement (defined as decrease in mean pulmonary artery pressure or improved cardiac output or improved PaO_2_/FiO_2_) with a dosage titrated up to 40 ppm.

Veno-venous ECMO (Cardiohelp, Getinge, Rastatt, Germany) was performed in cases of hypoxemia (PaO_2_/FiO_2_ < 100 mm Hg) refractory to advanced conservative treatment (prone position, optimized PEEP, and inhaled nitric oxide) or hypercarbia with severe respiratory acidosis and hemodynamic instability and/or right heart failure. Cannulation for veno-venous ECMO was either bifemoral, femoro-jugular, or via a jugular double lumen cannula (Avalon^®^, Getinge, Rastatt, Germany) depending on the individual patient’s anatomy. ECMO blood flow was adjusted to provide adequate oxygenation with a target of more than 50% of cardiac output. Sweep gas flow was adjusted to obtain normocarbia with ultra-protective ventilation (Vt = 4 mL/kg ideal body weight).

#### 2.3.2. Specific Treatment

Patients with confirmed influenza infection were treated with oseltamivir (75 mg po bid for ten days). Oseltamivir was initiated on admission to ICU if not already established prior to transfer to our department or continued to a total of ten days if begun previously. The treatment protocols for COVID-19 evolved over the course of the pandemic. Treatments are reported in Table 3.

Some patients were included in treatment trials [21]. Antibiotic therapy was withheld unless there were signs of bacterial infection (i.e., procalcitonin concentration in the serum >1 ng/mL, positive results on microbiological testing). Antibiotic therapy was reviewed at least bi-weekly by the local antibiotic stewardship team (microbiologists and intensivist) in all patients.

### 2.4. Statistic

Stata 16 (StataCorp LLC, College Station, TX, USA) was used for statistical analysis.

We compared baseline characteristics of the two groups using standardized differences. A standardized difference >0.1 suggested imbalance between groups [22]. A *t*-test was also performed with a *p* < 0.05 suggesting a significant difference.

To analyze further differences between groups, χ^2^ tests, Student *t* tests, and Mann–Whitney tests were used for categorical, symmetrically distributed continuous and non-normal continuous variables, respectively as indicated. All analyses were retrospective and with an exploratory intention. A *p* < 0.05 was considered significant.

## 3. Results

During the first wave of the COVID-19 pandemic (January–July 2020), the number of COVID-19 patients admitted to our ICU equaled the number of influenza patients in four consecutive flu seasons combined.

### 3.1. Baseline Characteristics of Patient Groups

Baseline Characteristics of the two groups are reported in Table 4.

Influenza infections were predominantly due to influenza A (90.6%), with 62% of Influenza A infections caused by the H1N1 type. 9.4% of influenza cases were caused by influenza B virus.

COVID-19 patients admitted to our ICU were significantly older than those admitted with influenza. Significantly more male COVID-19 patients than female ones were admitted to our ICU. The proportion of male patients among COVID-19 patients was higher than that of those admitted with influenza.

While the percentage of active smokers among the patients admitted with influenza (25%) was in concordance with the prevalence of smoking in Germany [23], there were significantly fewer active smokers amongst patients with COVID-19 (6.3%).

The BMI of influenza patients (30.2 kg m^−2^) was not significantly higher than that of patients admitted with COVID-19 (28.8 kg m^−2^).

The Charlson Comorbidity Index (CCI) was used to categorize preexisting conditions. It was not significantly different between influenza (2.53) and COVID-19 patients (2.48). A CCI of 2.5 suggests a 10-year survival of 85% based on the comorbidities [24].

The New Simplified Acute Physiology Score (SAPSII) [19] was assessed on admission to quantify severity of disease.

Initial disease severity was greater in COVID-19 patients with an average SAPSII on admission of 36.90 vs. 30.92 in influenza patients (*p* = 0.026). These result in predicted mortalities of 20% and 12%, respectively.

### 3.2. Clinical Endpoints

The clinical endpoints and the outcome of patients are reported in Table 5.

The mortality of COVID-19 patients was 34.3% and did not differ significantly from the mortality of influenza patients. Mortality was greater than predicted by the SAPSII value on admission in both cohorts.

**Figure 1:**
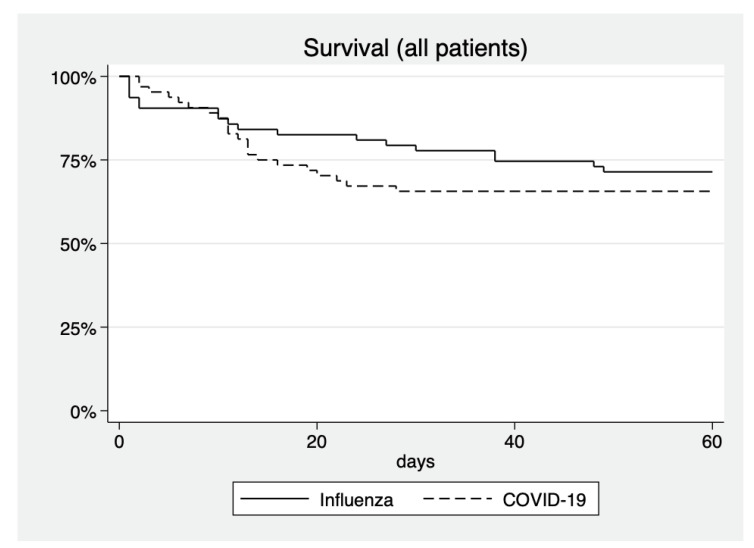
Survival curves of all patients treated in ICU. *p* = 0.568 for difference between groups.

**Figure 2:**
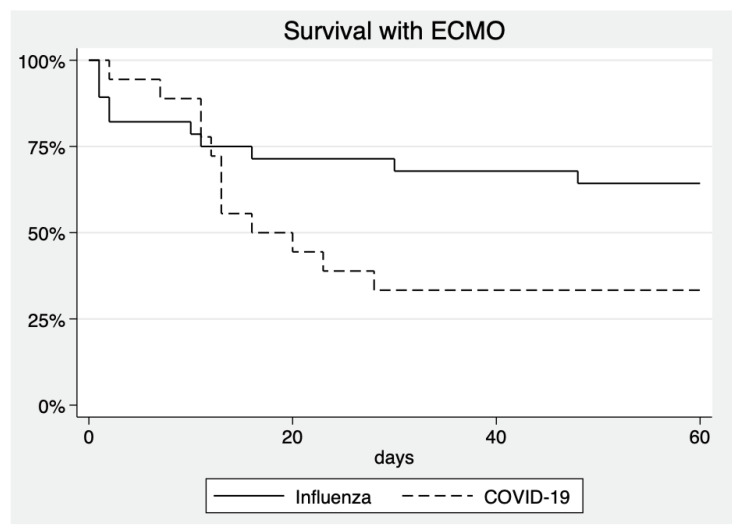
Survival curves of patients with any ECMO treatment during ICU stay. *p* = 0.055 for difference between groups.

45.3% of influenza patients were treated with ECMO, compared to 28.1% of COVID-19 patients (*p* = 0.044). Amongst patients treated with ECMO, mortality was 37.9% in influenza patients, compared to 66.7% in COVID-19 patients (*p* = 0.059). Mortality with and without ECMO is visualized in Figure 1 and Figure 2.

Influenza patients spent a mean of 23.1 days in ICU, relevantly but not significantly longer than those with COVID-19 (15.5 days; *p* = 0.0549).

Bacteremia occurred significantly more often during the treatment of influenza patients compared to patients treated with COVID-19.

Invasive aspergillosis was observed in 18.8% of influenza patients which is in accordance with the literature [25]. While aspergillosis was less frequent in COVID-19 patients (7.8%), this difference was not statistically significant (*p* = 0.063).

The Therapeutic Intervention Scoring System (TISS) [26] is routinely used for billing in intensive care in Germany, it was employed to quantify resource utilization in this study.

Both patient groups caused a similar workload expressed by TISS scores not being different between the groups.

## 4. Discussion

The impact of the COVID-19 pandemic on global health care systems was much greater than that of influenza [27] with more people infected, symptomatic, hospitalized, or critically ill than during recent flu seasons [28]. Yet, both diseases bear striking similarities: Both are caused by respiratory viruses with airborne transmission [29] prevalent in the community. Both have high rates of morbidity, place a heavy burden on healthcare systems and spread in pandemic events.

While our intensive care unit faces a large number of ARDS patients with influenza each season, the numbers treated during the COVID-19 pandemic were much higher.

With the onset of COVID-19 cases, influenza almost disappeared. While not part of this study, it should be noted that we did not observe a single case of influenza during the 2020/2021 season until this manuscript was submitted.

While the absolute number of patients being treated during the first wave of the pandemic was much higher than that of influenza patients treated during any previous flu season, there were many striking similarities.

The duration of stay in ICU was longer for patients with influenza (23.1 days vs. 15.5 days) without reaching statistical significance. Patients in both groups experienced prolonged treatments in ICU. With treatment algorithms being similar, this is not due to a more or less aggressive treatment in either disease. Especially with high numbers of patients with COVID-19 being admitted to ICU during the various waves of the pandemic, this finding is certainly relevant regarding resource utilization.

The mortality was not significantly different between both groups despite a markedly high mortality in COVID-19 patients treated with ECMO. There were 87.5% of influenza patients who required invasive ventilation. This was not significantly different from patients infected with SARS-CoV-2 (78.1%; *p* = 0.160). Influenza patients admitted to our ICU had similar comorbidities as expressed by the CCI (2.53) when compared to those treated with COVID-19 (2.48).

An important finding was the different features between patients presenting with influenza and those with COVID-19: COVID-19 patients were older and more likely to be male, which is in line with risk factors published previously [30,31,32]. A higher percentage was admitted from other hospitals.

The low prevalence of smokers among our COVID-19 patients is remarkable. This is in line with a few publications claiming lower rates of SARS-CoV-2 infections in countries with a high prevalence of smoking, a lower number of hospitalized current smokers than expected [33] and even leading to a controversial and probably premature suggestion for considering nicotine as a therapeutic option [34]. Nicotine acts in a similar fashion as the naturally occurring neurotransmitter acetylcholine on nicotinic acetylcholine receptors and may act as an anti-inflammatory agent [35]. Cholinergic signaling has been shown to influence the outcome of sepsis [36]. If these mechanisms play a role in the interaction of smoking and COVID-19 is beyond the scope of our study and a potential target for future examinations. However, this observation is contrary to most recent publications putting smokers at a higher risk for severe courses of COVID-19 [37].

While not reaching a significant difference to patients with influenza, mortality amongst COVID-19 patients requiring ECMO treatment is markedly high with approximately two-thirds of patients not surviving. This is higher than the results published by one register [38] with patients being much younger than our patients. Inclusion and exclusion criteria for ECMO vary by center and directly influence the outcome making results difficult to compare. ECMO mortality in COVID-19 patients is about doubled compared to the usual ECMO mortality rate at our center. Despite the high mortality in COVID-19 patients, we feel that ECMO remains a viable option in cases of severe ARDS and COVID-19 refractory to conservative treatment.

Mortality in both groups was markedly higher than predicted by the SAPSII score on admission to our ICU. This underlines that these patients are at risks for severe disease and adverse outcome and might influence decisions regarding ICU admission.

We observed a low incidence of aspergillosis in COVID-19 patients. This corresponds to data published from a large patient cohort [39]. Others published higher incidences [40,41] and COVID-19 associated pulmonary aspergillosis (CAPA) is being recognized as an entity [42]. Our study was conducted before the routine use of steroids emerged in the treatment of COVID-19 [43]. With steroid treatment being a risk factor for the development of invasive pulmonary aspergillosis [44], an increase in the incidence of CAPA with steroid use for treatment of COVID-19 can be imagined.

Our retrospective analysis has distinct strengths and shortcomings.

All patients were treated at a single center with a highly standardized protocol by a team experienced in treating ARDS and providing ECMO support.

Severe limitations are the small sample size and the evolution of COVID-19 therapy over the course of the pandemic. Initially, patients were treated with hydroxychloroquine following anecdotal reports from other countries. With studies showing the futility of such therapy and remdesivir becoming available on a routine basis, the latter was used as a standardized therapy. A few patients received reconvalescent plasma provided by the local department of transfusion medicine.

Two patients were included in a study examining the effect of sarilumab, an antibody targeting the interleukin-6 receptor.

All COVID-19 patients included were treated over a fairly short interval and are being compared to a historical control of influenza patients treated at the same facility over the previous years. While this is a potential shortcoming, the virtual disappearance of influenza during the COVID-19 pandemic [45] renders a comparison of patients treated simultaneously impossible.

All patients admitted during the observational period were included. This created a relevant inhomogeneity amongst the groups which might constitute a limitation.

Our study represents patients being treated at a single institution’s ICU. The overall outcome of the disease might be different with morbidity and mortality outside of ICU or at different institutions not being examined in the present analysis.

## 5. Conclusions

COVID-19 and influenza patients have a similar outcome in ICU in cases of severe disease requiring invasive ventilation. If ECMO is necessary, mortality amongst patients with COVID-19 is high. COVID-19 patients were likely to be older and male. Active smokers were less prevalent among COVID-19 patients.

COVID-19 is a frequent disease with infection rates at times exceeding 100 per 100,000 people per week and with about a third of patients requiring hospital treatment eventually being admitted to ICU. The course of these patients is similar to those with severe influenza with long courses in ICU and a substantial proportion of patients requiring ECMO treatment. Combined with the high number of patients, the findings explain the exhaustion of health care systems observed in many areas of the world during the pandemic.

## Figures and Tables

**Table 1:** Patient population with admission periods (top) and virus identified (bottom).

	Influenza (*n* = 64)	COVID-19 (*n* = 64)
2016/2017 flu season	27	0
2017/2018 flu season	11	0
2018/2019 flu season	17	0
2019/2020 flu season	9	0
January 2020–July 2020	n/a	64
Influenza A (non H1N1)	22	
Influenza A (H1N1)	36	
Influenza B	6	
SARS-CoV-2		64

**Table 2:** Standardized diagnostics and treatment protocol.

Standardized Protocol for ARDS
**Diagnostics on Admission**
- Invasive Monitoring with arterial line and pulmonary artery catheter
- Transesophageal echocardiography
- Computed Tomography of chest and abdomen
- Sampling for micriobiological testing ▪ Blood cultures ▪ Bronchioalveolar lavage ▪ Gram stain and immediate microscopy ▪ Cultures ▪ PCR (multiplex for common causes of pneumonia) ▪ PCR (*M. tuberculosis, P. jirovecii, L. pneumophila*) ▪ PCR (*Aspergillus fumigatus*) ▪ PCR (viral panel, including SARS-CoV-2 in 2020) Samples from 2019 tested retrospectively for SARS-CoV-2 ▪ Galactomannan ▪ Urine ▪ Culture ▪ Antigen testing for *L. pneumophila* and *S. pneumoniae* ▪ Nasal and rectal swab ▪ Screening for MRSA, VRE, and resistant gram-negative pathogens
**Therapy**
- Ventilatory support: ▪ Non invasive ventilation ▪ Indications for intubation • Severe dyspnea and/or exhaustion • Respiratory rate persisting > 30 min^−1^ ▪ Invasive Ventilation ▪ BIPAP-mode ▪ I:E with expiration sufficient to prevent air trapping ▪ FiO_2_ adjusted to keep SpO_2_ > 90% ▪ Best PEEP trial with PEEP > lower inflection point in low flow P/V-loop ▪ Tidal volume set to 6 mL kg^−1^ (ideal body weight) ▪ Driving pressure < 15 mbar ▪ Prone position (135° with the better lung down) for 16 h/d ▪ Nitric oxide (NO) trial up to 40 ppm • Criteria for positive response: - Drop in mean pulmonary artery pressure - Increase in cardiac output - Improved P/F ratio ▪ Percutaneous dilatational tracheostomy ▪ Weaning failure ▪ Prolonged (>1 week) invasive ventilation
- Antibacterial Therapy ▪ Antibiotics only with elevated procalcitonin or positive results in microbiological testing
- Antiviral therapy ▪ Changed during pandemic, see separate table
- ECMO ▪ P/F ratio < 100 despite optimized treatment ▪ Hypercarbia with pH < 7.2 or hemodynamic instability ▪ Inability to provide driving pressure < 15 mbar and/or Vt < 6 mL kg^−1^ ▪ Rapid progres

PCR—polymerase chain reaction; MRSA—methicillin-resistant Staphylococcus aureus. VRE—vancomycin-resistant enterococci; I:E—inspiration:expiration ratio; P/V—pressure/volume.

**Table 3:** Specific treatments for viral infections.

Specific Treatment	*n*
**Influenza—patients**	
- Oseltamivir	60
**COVID-19—patients**	
- Remdesivir	12
- Hydroxychloroquine	26
- Reconvalescent-Plasma	8
- Sarilumab/Placebo (trial)	2

**Table 4:** Baseline Characteristics between groups. Mean and 95%-confidence intervals or absolute number and percentage are reported.

	Influenza (*n* = 64)	COVID-19 (*n* = 64)	Std Diff ^#^	*p*-Value
Age [years]	54.1 (49.8; 58.4)	60.1 (56.9; 63.3)	0.39	0.0211 ^§^
Sex [male]	41 (64%)	54 (84%)	0.40	0.0292 ^$^
BMI [kg m^−2^]	30.2 (27.9; 32.5)	28.8 (25.2; 32.4)	0.18	0.5669 ^§^
Charlson Comorbidity Index	2.53 (1.93; 3.13)	2.48 (2.00; 2.97)	0.02	0.9847 ^§^
Active Smoker	16 (25%)	4 (6.3%)	0.53	0.0037 ^$^
Transfer from other hospital	39 (60.9%)	43 (67.2%)	0.12	0.4651 ^$^
SAPS II on admission	30.92 (26.56; 35.27)	36.90 (33.89; 39.91)	0.40	0.0261 ^§^

^#^ A standardized difference >0.1 suggests an imbalance between the groups. ^§^ *t*-test. ^$^ χ^2^ test. BMI—body mass index. SAPSII—Simplified Acute Physiology Score II.

**Table 5:** Clinical endpoints of patients treated in ICU with severe influenza compared to those treated with severe SARS-CoV-2 Infection (Covid19). A *p* < 0.05 was considered significant (*t*-test for independent samples).

	Influenza (*n* = 64)	COVID-19 (*n* = 64)	*p*-Value
Mortality	19 (29.7%)	22 (34.3%)	0.568
Any ECMO	29 (45.3%)	18 (28.1%)	0.044
Mortality with ECMO	11/29 (37.9%)	12/18 (66.7%)	0.055
Invasive ventilation	56 (87.5%)	50 (78.1%)	0.160
Time in ICU [days]	23.1 (15.9; 30.3)	15.5 (12.3; 18.7)	0.0549
Any dialysis (CVVHD) in ICU	32 (50.0%)	24 (37.5%)	0.131
Any bacteremia	46 (71.9%)	33 (51.6%)	0.023
Any invasive aspergillosis	12 (18.8%)	5 (7.8%)	0.063
TISS points per day	15.50 (12.77; 18.21)	17.14 (13.77; 20.51)	0.448

ECMO—Extra corporeal membrane oxygenation; CVVHD—continuous veno-venous hemodialysis; ICU—intensive care unit; TISS—Therapeutic intervention scoring system.

## Data Availability

The data presented in this study are available on request from the corresponding author. The data are not publicly available since they contain individual patients’ data and public availability was not consented to.
